# Analysis of immune status in gastric adenocarcinoma with different infiltrating patterns and origin sites

**DOI:** 10.3389/fimmu.2022.978715

**Published:** 2022-08-23

**Authors:** Nana Zhang, Depu Wang, Xiaoyan Hu, Guanjun Zhang, Zhuoqun Li, Yan Zhao, Zhijun Liu, Yili Wang

**Affiliations:** ^1^ Institute of Regenerative and Reconstructive Medicine, Med-X Institute, First Affiliated Hospital of Xi’an Jiaotong University, Xi’an, China; ^2^ Institute for Cancer Research, School of Basic Medical Science, Health Science Center of Xi’an Jiaotong University, Xian, China; ^3^ Department of Science and Technology, The First Affiliated Hospital of Xi’an Jiaotong University, Xian, China; ^4^ Department of Pathology, The First Affiliated Hospital of Xi’an Jiaotong University, Xian, China

**Keywords:** tumor infiltration pattern, tumor-infiltrating lymphocytes, tumor origin site, gastric adenocarcinoma, immune status

## Abstract

Tumor infiltration pattern (INF) and tumor origin site were reported to significantly affect the prognosis of gastric cancer (GC), while the immune status under these contexts is not clear. In this study, we correlated the density and phenotype of tumor-infiltrating lymphocytes (TILs) with INF and the tumor origin site to reflect the biological behavior of tumors from a new perspective and also determined their effects on overall survival (OS) and other related clinicopathological features in archival samples of 147 gastric cancers with 10-year follow-up data. We found that the INFc growth pattern (an invasive growth without a distinct border) of GC lacked immune cell infiltration, particularly the cytotoxic T cells and their activated form. It is also significantly associated with an unfavorable prognosis (*P* < 0.001) and proximal site (*P* = 0.001), positive lymph node metastasis (*P* = 0.002), and later tumor–node–metastasis stage (*P* < 0.001). Moreover, the density and sub-type of TILs infiltration were significantly different in disparate differentiated areas for the tumor tissue with INFb. Compared with distal gastric cancer, proximal gastric cancers were prone to grow in an INFc pattern (*P* = 0.001) and infiltrated with fewer TILs, experiencing a shorter survival time (*P* = 0.013). Multivariate analysis showed that only the INF and the density of TILs were demonstrated to be the independent prognostic factors of OS for the GC. We concluded that GC with an aggressive growth pattern arising from proximal sites always had a weak immune response and resulted in a poor prognosis. The interaction between them and their synergistic or antagonistic effects in the development of tumors need to be further studied. This study opens up a new perspective for research on the biological behavior of the tumor.

## Introduction

Gastric cancer (GC) is one of the most common cancers worldwide for both male and female individuals (1). Many clinicopathological elements were reported to influence the patients’ survival, such as tumor–node–metastasis (TNM) stage, histopathological type, and genetic factors (2, 3). Even The Cancer Genome Atlas project has also involved GC classification by displaying four sub-types, *i*.*e*., tumors positive for Epstein–Barr virus, microsatellite unstable tumors, genomic stable tumors, and tumors with chromosomal instability, which have corresponding molecular profiles and are aimed at potential targeted therapies (4).

The tumor originating sites and growth patterns as essential pathological parameters in gastric cancer and also their clinical significance have been often described (5, 6). Tumor infiltration patterns (INFs) were classified into three according to the Japanese Classification of Gastric Carcinoma: INFa, INFb, and INFc. The INFa group exhibits expanding growth and a distinct border with the surrounding tissue and INFc is described as displaying infiltrating growth and an indistinct border with the surrounding tissue, while INFb falls between INFa and INFc (7). Their features were shown to be valuable in predicting the prognosis and recurrence pattern in advanced GC (6) and so were the primary sites of GC, for instance, the primary GC arising in the upper third of the stomach, including the cardia or gastroesophageal junction, usually addressed as proximal gastric cancer (PGC), was reported to be associated with a worse prognosis compared with distal cancers (DGC) originating from the rest of the stomach (8). Moreover, the incidence of adenocarcinoma at the antrum or distal stomach has decreased, whereas that of the proximal type has increased in most developed countries (9, 10). There are discrepancies between PGC and DGC in terms of biological behaviors and etiologic factors. PGC shows demographic and pathological features typical of Barrett’s-related esophageal adenocarcinoma and is not associated with severe forms of gastritis characterized by atrophy and/or intestinal metaplasia and/or a *Helicobacter pylori* infection, which was proven to be a key factor in adenocarcinomas of the distal stomach (11–13). For the anatomical structure of PGC, the serosa is partially developed, and it is prone to be diagnosed at a more advanced stage, indicating an unfavorable prognosis (14). It can be concluded that PGC possesses a more aggressive biological behavior more frequently associated with deeper gastric wall infiltration, lymph node involvement, and lymphatic vessel invasion (15). It has been noted that a GC with a different INF is reflected by its aggressive abilities. The INFc growth pattern exhibited more aggressive and more budding tumor cells, but not the INFa pattern, and the budded tumor cells harbored some stemness properties and epithelial–mesenchymal transition phenotypes (16).

Up to now, few studies focused on the contact of the tumor originating site and INF, both of which were specifically behavioral characteristics of GC and affect the patients’ prognosis. Furthermore, nearly no study has involved local immunity state with tumor originating site and INF. Nevertheless, we wonder if the histological heterogeneity of GC in INF and tumor arising sites could be more informative relative to the local immune status, *i*.*e*., GC with different INF and primary sites could underlie the privileged immunobiological behavior of the tumor cells and is of great importance to understand the influence of the tumor microenvironment on cancer development and evolution. It has been well documented that the presence of tumor-infiltrating lymphocytes (TILs) correlated to the patients’ outcomes (17, 18). Specifically, the prognosis of tumor patients could be predicated on the type, density, and location of immune cell infiltration, as the different sub-types of TILs could affect the behavior of the tumor, inhibiting or promoting neoplastic progression (19, 20). It would be reasonable to deem that the primary sites of GC and different INFs could create a particular immune microenvironment and influence a patient’s outcome. Therefore, we performed a study of 147 patients with gastric adenocarcinoma with complete 10-year follow-up data to evaluate the association of the tumor with different cancer arising sites and INF and then analyzed their corresponding immune status, which may contribute to the clinical diagnosis and treatment of gastric cancers as well as explain the biological behaviors of tumor cells comprehensively.

## Materials and methods

### Patients and specimens

A total of 147 primary gastric cancer patients with complete 10-year follow-up data (116 male and 31 female patients; mean age, 62.3 years) between 2001 to 2003 at the Department of Pathology of First Affiliated Hospital of Xi’an Jiaotong University were recruited. The patients underwent a curative total or subtotal gastric resection along with regional lymphatic dissection, without distant metastasis in any patient upon preoperative examination. The data collected for analysis included age, gender, Lauren classification, TNM stage, histological differentiation, tumor location, tumor size, and lymph node involvement of the patients. The detailed information is presented in **Table 1** of our previous study (21). All specimens were fixed in 10% buffered formalin and embedded in paraffin wax. The maximal invasive margin was selected and sliced into 4-μm sections to conduct hematoxylin and eosin (H&E) and immunohistochemistry (IHC) staining. Five serial sections of each paraffin-embedded tumor block were cut—one for H&E to inspect the INF and four for IHC to detect the TILs.

**Table 1:** Association of INF with clinicopathologic parameters.

Clinicopathologic parameters	No. of cases (%)	INFc	INFa+b	χ2 value
Tumor arising site				
Proximal	38 (27.9)	24	14	0.001
Distal	109 (72.1)	35	74	
Age (years)				
≤60	79 (53.7)	26	53	0.329
>60	68 (46.3)	33	35	
Gender				
Female	31 (21.1)	18	13	0.022
Male	116 (78.9)	41	75	
Tumor size (cm)				
≤4 cm	86 (58.5)	37	49	0.231
>4 cm	61 (41.5)	22	39	
Lymph nodes involvement				
Negative	62 (42.2)	16	46	0.002
Positive	85 (57.8)	43	42	
No,of positive Lymph nodes				
≤5	108 (73.5)	33	75	< 0.001
>5	39 (26.5)	26	13	
TNM stage				
IA-IB	39 (26.5)	5	34	< 0.001
IIA-IIB	40 (27.2)	15	25	
IIIA-IIIC	68 (46.3)	39	29	
IV	0 (0)	0	0	
Grade				
G1	3 (2.0)	1	2	0.958
G2	58 (39.5)	22	36	
G3	70 (47.6)	29	41	
G4	16 (10.9)	7	9	
Lauren classification				
Intestinal type (IT)	86 (58.5)	26	60	8.703
Diffuse type (DT)	35 (23.8)	18	17	
Mixed type (MT)	26 (17.7)	15	11	

### Classification of tumor location

According to the criteria of the Japanese Gastric Cancer Association (7), the tumor location was divided into two groups, *i*.*e*., proximal gastric cancer (PGC) and distal gastric cancer (DGC), by reviewing the clinicopathological data. PGC was considered when the tumor arose in the upper third of the stomach, including the cardia or gastroesophageal junction, which is up to the crossing line between the left gastric artery and the end of the left gastroepiploic artery. The tumors below this crossing line were considered DGC.

### Assessment of tumor infiltrating pattern

**Figure 1:**
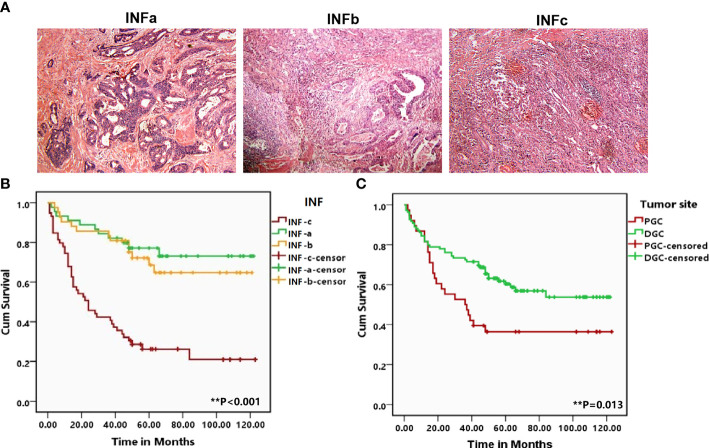
**(A)** Representative patterns of the three types of tumor infiltrating growth (INF) pattern of INFa, INFb, and INFc (×100) in the H&E staining slides. **(B, C)** Corresponding Kaplan–Meier survival curves for proximal gastric cancer (PGC) and distal gastric cancer as well as the different types of INF, respectively. The tumor from the PGC (*P* = 0.013) and infiltrating with INFc (*P* < 0.001) suffered a shorter overall survival. The degree of difference is expressed by the asterisk symbols: **P < 0.001 and *P < 0.05.

The INF types were determined by observing sections stained with H&E, strictly according to the Japanese Classification of Gastric Carcinoma (7). The tumor growth pattern was classified as INFa (expansive growth having a distinct border with the surrounding tissues), INFb (intermediate type between INFa and INFc), and INFc (infiltrative growth having no distinct border with the surrounding tissues) (**Figure 1A**). Two expert pathologists reviewed the sections to confirm the diagnosis.

### Assessment of differentiation differences in the same section

According to the differentiation of tumor cells in different regions of the sectioned tissue of INFb, the tumor tissue was divided into well-differentiated and poorly differentiated regions. Well-differentiated areas are those where the tumor cells were characterized by cohesive cells which form gland-like structures. Poorly differentiated areas are those where tumor cells infiltrate the stroma as a single cell or small cell cluster, leading to a population of non-cohesive, scattered tumor cells.

### Immunohistochemistry

Immunohistochemical staining was carried out using the streptavidin–biotin–peroxidase method. The mouse monoclonal primary antibodies used were anti-human CD8 (DakoCytomation, Glostrup, Denmark; 1:100 dilution), anti-human granzyme B (Novocastra, Newcastle, UK; 1:100), anti-human OX40 (Novocastra; 1:30), and anti-human Foxp3 (Abcam, Cambridge, UK; 1:50) to identify the lymphocyte immunophenotype. Normal lymph node tissue was used for positive controls. Sections were deparaffinized in xylene and rehydrated in a graded series of ethanol. Endogenous peroxidase activity was blocked by 10-min incubation with 3% hydrogen peroxide in methanol. After washing in TBST, antigen retrieval was done by heat-induced epitope retrieval methods for 1 min and 30 s in citric buffer (pH 6.0), then saturated with 10% normal goat serum for15 min, and then incubated with a primary antibody at 4°C overnight. Subsequently, sections were incubated with Dako EnVision (DakoCyomation, Denmark) for 30 min at room temperature. Color development was visualized with freshly prepared diaminobenzidine (DAB)–chromogen for 5 min. The slides were counterstained with hematoxylin and mounted on coverslips. For the sake of showing a clear image of TILs and INF on one slide no matter at high or low magnification, we stained the CD8+TILs and tumor cells in one slide with the double-IHC staining. Similar to the IHC, after detecting the CD8+T cells by DAB, another incubation was performed with anti-CK (AE1 + AE3; Abcam, Cambridge, UK; prediluted) for 2 h at room temperature, followed by an application of 5-bromo-4-chloro-3-indolyl phosphate for 10 min and counterstaining with nuclear fast red for 3 min. The tumor cells were stained purple–blue, and the CD8+T cells were colored brown.

### Evaluation of positive TILs

The counting of positive TILs was performed by the classical point counting method as described by Anderson (22). A 100-point ocular grid was used at ×400 magnification under a microscope (Olympus Optical Co., Ltd., Tokyo, Japan). Excluding the influence of subjective factors, the immune cell was observed in 10 fields with the most abundant positive cell distribution for each tissue sample bypassing the lymph follicle and the normal tissue on the slides. As for the limited fields of the well-differentiated and poorly differentiated areas in one slide with INFb, only five fields with the most abundant positive cells were selected. The counted fields only included cancer cell nests and surrounding tissue stroma, within the tumor tissue. The number of positive TILs was counted twice for each slide, and the mean value was calculated for each case as the final count. The cases were divided into TIL-high and TIL-low groups according to the sub-type of the TIL median for further analysis with the INF and tumor site.

### Statistical analysis

SPSS 13.0 for Windows (SPSS, Chicago, IL, USA) was used for the statistical analysis. The distribution difference of the four sub-types of TIL according to the INF and tumor location as well as different regions of differentiation was analyzed by one-way ANOVA and independent-samples *T*-test, respectively. Correlations of the INF and TILs and tumor location, as well as other clinicopathological variables, were determined by the chi-square test. The Kaplan–Meier method was used to estimate overall survival, and survival was analyzed by the log-rank test based on INF, TILs, and tumor location. Univariate and multivariate analyses of the three factors and of the clinicopathological features were performed using the Cox proportional hazard regression model. *P <*0.05 was regarded as significant in all of the analyses.

## Results

### The relationship of INF and tumor origin site and their association with pathological parameters in gastric adenocarcinoma

The results of the correlation analysis showed that INF and tumor origin site were statistically correlated to each other (*P* = 0.001). Moreover, 63.16% (24/38) cases of tumors originating from the proximal site are growing with INFc pattern, and 67.89% (74/109) cases of tumors arising from the distal site are infiltrating with INFa or INFa pattern. It indicated that PGC tends to grow in a malignant infiltrative pattern (INFc), whereas DGC tends to grow in a relatively benign infiltrating pattern (INFa + INFb) (**Table 1**). For the relationship between INF and other pathological parameters, female patients are more prone to appear INFc (*P* = 0.022). Tumor from the proximal site was significantly related to tumors with INFc (*P* = 0.001) presence of lymph node metastasis (*P* = 0.002), and a higher number of positive lymph nodes were more frequent in patients with INFc tumors than in those with INFa/b tumors (*P* < 0.001). Additionally, tumors with INFc were significantly related to a later TNM stage (*P* < 0.001) and a mixed type of Lauren classification (*P* = 0.013). There was no significant difference in tumor differentiation and patients’ age between INFa/b and with INFc (**Table 1**).

The comparisons on the relationships of age, gender, tumor size, number of positive lymph nodes, and Lauren classification between PGC and DGC showed no statistical difference, while a larger tumor size (*P* = 0.072), a higher number of positive lymph nodes (*P* = 0.095), and Lauren classification (*P* = 0.087) tend to be associated with the tumor location. PGC was statistically associated with a later TNM stage (*P* < 0.001) and positive lymph node metastasis (*P* = 0.007) (**Table 2**).

**Table 2:** Association of originating site of GC with clinicopathologic parameters.

clinicopathologic parameters	No. of cases (%)	Proximal GC	Distal GC	χ2 value
Age (years)				
≤60	79 (53.7)	21	59	0.325
>60	68 (46.3)	18	50	
Sex				
Female	31 (21.1)	6	25	0.352
Male	116 (78.9)	32	84	
Tumor size (cm)				
≤4 cm	86 (58.5)	24	62	0.072
>4 cm	61 (41.5)	14	47	
Lymph nodes involvement				
Negative	62 (42.2)	9	53	0.007
Positive	85 (57.8)	29	56	
No, of positive Lymph nodes				
≤5	108 (73.5)	24	84	
>5	39 (26.5)	14	25	0.095
TNM stage				
IA-IB	39 (26.5)	4	35	< 0.001
IIA-IIB	40 (27.2)	6	34	
IIIA-IIIC	68 (46.3)	28	40	
IV	0 (0)	0	0	
Pathological grade				
G1	3 (2.0)	0	3	0.609
G2	58 (39.5)	17	41	
G3	70 (47.6)	18	52	
G4	16 (10.9)	3	13	
Lauren classification				
Intestinal type (IT)	86 (58.5)	28	58	0.087
Diffuse type (DT)	35 (23.8)	6	29	
Mixed type (MT)	26 (17.7)	4	22	

### The prognostic effect of INF and tumor origin site on GC patients

Log-rank test showed that GC in the proximal site experienced a much shorter survival time (*P* = 0.013; **Figure 1B**). Moreover, the prognosis of the patients with INFc tumor was significantly worse than that with INFa or INFb in all cases (*P* < 0.001; **Figure 1C**). Univariate and multivariate analyses revealed that INFc was an independent risk prognostic factor of the OS of GC patients (**Table 3**). Additionally, INFc (HR = 3.079, *P* < 0.001), positive lymph node metastasis (HR = 3.883, *P* = 0.004), and diffused type of Lauren classification (HR = 2.647, *P* = 0.006) were found to be independent risk prognostic factors for GC patients. Only a higher number of TILs (HR = 0.515, *P* = 0.019) was found to be a favorable prognostic factor for GC patients (**Table 3**).

**Table 3:** Univariate and multivariate analyses of prognostic factors for survival of gastric cancer patients.

Variables	Categories	Univariable analysis		Multivariable analysis	
		HR (B95%CI)	*P*	HR (95%CI)	*P*
INF	INFc *vs* INFa+b	4.288 (2.593, 7.092)	<0.001	3.079 (1.683, 5.632 )	0.009
Age (years)	>60 *vs* ≤60	2.150 (1.266, 3.711)	0.008	1.484 (0.706, 3.119)	0.297
Gender	Male *vs* female	1.286 (0.689, 2.400)	0.430	1.328 (0.555, 5.136)	0.631
Tumor size (cm)	>4 *vs* ≤4	1.860 (1.152, 3.005)	0.011	2.519 (0.921, 4.464)	0.202
Tumor originating site	PGC *vs* DGC	1.858 (1.127, 3.064)	0.015	1.331 (0.755, 2.348)	0.323
Lymphnode metastasis	Positive *vs* negative	7.870 (3.884, 15.945)	<0.001	3.883 (1.545, 9.761)	0.004
No,of positive positive lymph nodes	>5 *vs* ≤5	4.243 (2.608, 6.904)	<0.001	0.902 (0.437, 1.862)	0.781
TNM stage	IIA-IIB *vs* IA-IB	4.453 (1.488, 13.322)	0.013	1.546 (0.426, 5.609)	0.508
	IIIA-IIIC *vs* IA-IB	12.023 (4.321, 33.458)	0.002	2.305 (0.622, 8.542)	0.211
Tumor grade	G2 *vs* G1	0.00 (0.000, 2.2E26)	0.969	0.000 (0.000, +∞)	0.978
	G3 *vs* G1	0.607 (0.290, 1.271)	0.185	1.713 (0.697, 4.215)	0.241
	G4 *vs* G1	0.823 (0.406, 1.667)	0.588	1.458 (0.677, 3.140)	0.335
Lauren classification	Diffuse type *vs* Intestinal type(IT)	2.196 (1.283, 3.762)	0.004	2.647 (1.327, 5.279)	0.006
	Mixed type *vs* IT	1.573 (0.825, 3.000)	0.169	1.460 (0.697, 3.061)	0.316
Density of TILs	High *vs* low	0.404 (0.244, 0.671)	<0.001	0.515 (0.296, 0.897)	0.019

HR: Hazard ratio, CI: Confidence interval, PGC: Proximal gastric cancer, DGC: Distal gastric cancer, INF: tumor infiltrating pattern.

### The immune status in gastric tumor originating from different sites and its prognostic value

**Figure 2:**
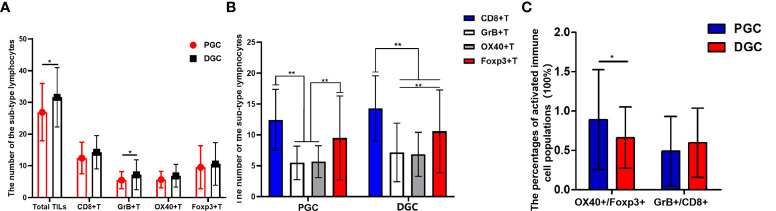
**(A, B)** Graphs showing the four sub-types of tumor-infiltrating lymphocytes (TILs) distribution in proximal gastric cancer (PGC) and distal gastric cancer (DGC). The total number of TILs (*P* = 0.033) and the GrB+T (*P* = 0.003) cell infiltrates were significantly attenuated in PGC **(A)**. The CD8+T cells possess a numerical advantage in DGC (*P* < 0.001) as for the investigated sub-type of immune cells. The number of Foxp3+T cells was also quantitatively superior to OX40+T (*P* < 0.001) and GrB+T (*P* < 0.001) cells in DGC and PGC **(B)**.The functional Treg cell (OX40+/FOXP3+) percentage was significantly higher in the PGC compared with that in DGC (*P* = 0.009) **(C)**. The degree of difference is expressed by the asterisk symbols: **P < 0.001 and *P < 0.05.

After clarifying the relationship between INF and tumor origin site, we further analyzed the immune status of GC tissues with different INF and originating sites to better understand their current impact on GC patients’ prognosis. The CD8+ T cells possess an anti-tumor effect. The Foxp3+ regulatory cells (Tregs), playing a critical role in immune tolerance and deficiency of anti-tumor immunity, were often used as a negative antitumor parameter. Therefore, the subset of TILs in our study contained CD8+ cytotoxic T cell and Foxp3+ Treg, supplemented with their activated form (GrB+T and OX40+T). In this cohort, 38 cases were adenocarcinomas of PGC, and 109 cases were in the distal stomach. Overall, the lymphocyte infiltrates in PGC tissue were relatively less than those in the distal site of GC tissue, although without statistical significance. Compared with DGC, the total number of TILs (*P* = 0.033) and the GrB+T (*P* = 0.003) cell infiltrates were significantly attenuated in PGC (**Figure 2A**), and the number of CD8+T and OX40+T cells were with an obvious tendency to be infiltrated less in the PGC group (PGC *vs*. DGC: CD8+T, 12.447 ± 4.941 *vs*. 14.294 ± 5.267, *P* = 0.061; OX40+T, 5.658 ± 2.581 *vs*. 6.844 ± 3.567, *P* = 0.062). The infiltration of regulatory T cells (Foxp3+T) was not significantly different between the two groups. Additionally, CD8+T cells possess a numerical advantage in both DGC (*P* < 0.001) and PGC among the investigated sub-types of immune cells, although without statistical significance in PGC. The number of Foxp3+T was also quantitatively superior to OX40+ (*P* < 0.001) and GrB+T (*P* < 0.001) cells in DGC and PGC (**Figure 2B**). We further analyzed the relative percentages of activated immune cell populations (GrB+/CD8+ and OX40+/FOXP3+) in the tumor tissue from the different originating sites. The results showed that the functional Foxp3+T cell percentage was significantly higher in PGC compared with that in DGC (*P* = 0.009), and there was no statistical significance between PGC and DGC for the percentages of the activated immune type of CD8+T cells (**Figure 2C**).

### The immune status in the gastric tumor of different INFs

**Figure 3:**
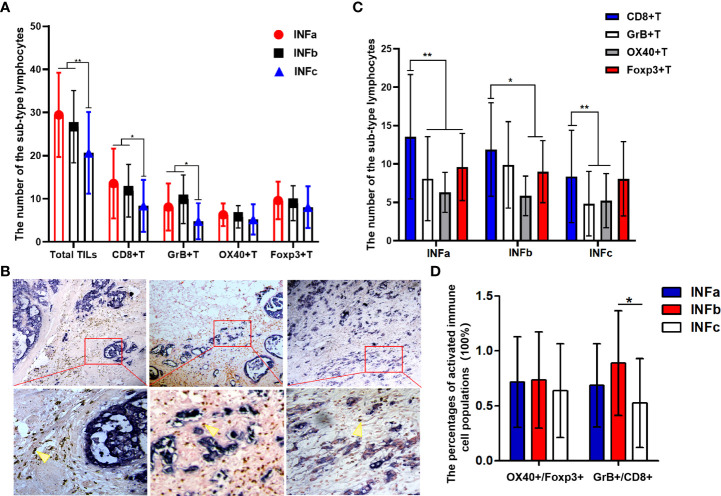
**(A, C)** Graphs showing the four sub-types of tumor-infiltrating lymphocyte distribution in cancer tissues with different types of tumor infiltration pattern (INF). The number of total immune cell infiltrates was less in INFc than that in INFa (*P* < 0.001) or INFb (*P* = 0.001) **(A)**. The number of CD8+T cells occupied a quantitatively dominant position (*P* = 0.001) in the INFa cases **(C)**. Representative double-immunohistochemistry staining for the tumor cells (purple–blue) and CD8+T cells (brown, yellow arrow) in gastric cancer tissue with different types of INF, and the lower pictures (×400) are the corresponding enlargement of the local area (red, rectangular) for the upper pictures (×100) **(B)**. The relative percentage of activated immune cell populations for CD8+ (GrB+/CD8+) is significantly higher in the INFb group compared with that in INFc (*P* = 0.02) **(D)**. The degree of difference is expressed by the asterisk symbols: **P < 0.001 and *P < 0.05.

There were 46 patients in INFa, 42 in INFb, and 59 in INFc who were among these 147 GC samples. The TILs in different INF exhibited a significant and uneven distribution (**Figure 3A**). In general, the number of total immune cell infiltrates was less in INFc than that in INFa (*P* < 0.001) or INFb (*P* = 0.001) pattern, whereas there was no significant difference between INFa and INFb for the number of total TILs. When the subsets of TILs were taken into consideration, the number of CD8+T (**Figure 3B**), GrB+T, and OX40+T cells did not show a significant difference between the cases of INFa and INFb patterns, but their infiltration in the cases of INFc was significantly less than those in the cases of INFa (CD8+T, *P* < 0.002; GrB+T, *P* = 0.001; and OX40+T, *P* = 0.001) and INFb (CD8+T, *P* = 0.011; GrB+T, *P* < 0.001; and OX40+T, *P* = 0.009) patterns. The infiltration of effector Th cells (OX40+) in cancer tissue with INFa tended to be more than that in INFc (INFa *vs*. INFc: 6.304 ± 2.615 *vs*. 5.220 ± 3.519, *P* = 0.083), but there was no significant difference in its distribution between INFa and INFb. As for the regulatory T cell infiltration, there was no significant difference among the three infiltrating patterns. Additionally, the number of CD8+T cells occupied a quantitatively dominant position (*P* = 0.001) in the INFa cases, but not in the INFb and INFc cases. The infiltration of CD8+T cells was significantly higher than the OX40+ (*P* < 0.001) and GrB+T cells (*P* < 0.001) but without advantages on Foxp3+T cells in INFc cases (**Figure 3C**). Moreover, the relative percentages of activated immune cell populations for the CD8+T cells (GrB+/CD8+) were significantly higher in the INFb group compared with the INFc group (*P* = 0.02), while OX40+/FOXP3+ did not show any statistical significance among the three INF groups (**Figure 3D**).

### The TILs infiltration difference in GC tissue with differentiation differences

**Figure 4:**
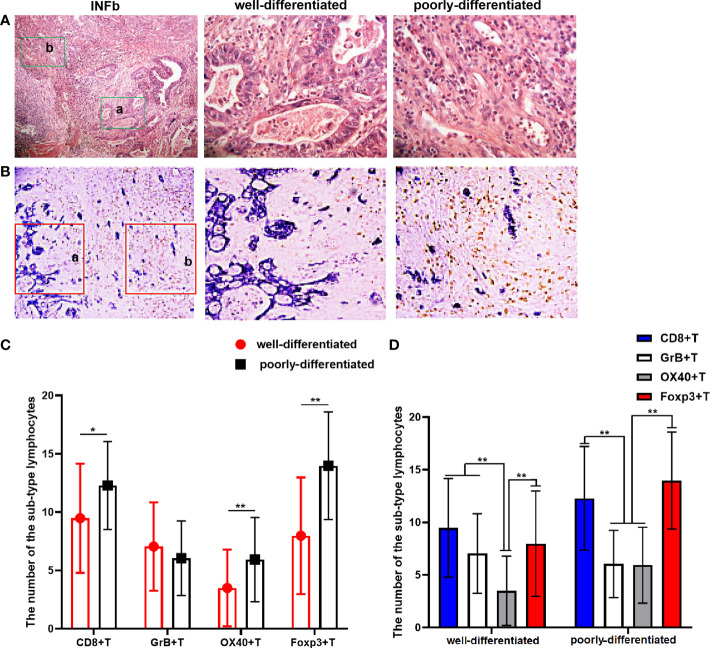
**(A)** The H&E staining result shows that there are significant differentiation differences regions in gastric cancer (GC) tissue with INFb growth pattern. The latter two pictures (×400) are local magnifications of the a and b (green rectangular) regions in the first picture (×100). **(B)** Double-immunohistochemistry staining representative image for CD8+T cells’ (brown) distribution in GC tissue with INFb. The latter two pictures (×200) are their corresponding enlargement of the local area (red rectangular) for the first pictures (×100) and represent the well and poorly differentiated region, respectively. **(C, D)** Graphs showing the four sub-types of tumor-infiltrating lymphocyte distributed differently according to the cancer cell differentiation in GC tissues with INFb. The degree of difference is expressed by the asterisk symbols: **P < 0.001 and *P < 0.05.

In gastric cancer sections of 42 cases with an infiltration pattern of INFb, there were distinct differentiation differences formed by tumor cells with different differentiation grades, which can be classified into well-differentiated and poorly differentiated areas (**Figure 4A**). We further analyzed the infiltration difference of the investigated sub-types of TILs in areas with different differentiation grades in the cancer tissues of INFb. The results showed that the number of Foxp3+ (*P* < 0.001), OX40+ (*P* = 0.001) and CD8+ T (*P* = 0.008) lymphocytes in poorly differentiated areas was significantly higher than that in the well-differentiated areas of the tumor, respectively (**Figures 4B, C**). Moreover, the dominance order of the four types in the well-differentiated areas was as follows: CD8+ > Foxp3+ > GrB+ > OX40+T lymphocytes, while in the poorly differentiated areas, this was as follows: Foxp3+ > CD8+ > OX40+ > GrB+ T lymphocytes (**Figure 4D**), although not all sub-types are significantly different from each other. These results indicated that tumor cells with different differentiation grades have various abilities to recruit different sub-types of lymphocytes, resulting in the discrepancy of the type and number of recruited lymphocytes despite being under the same immunological background.

## Discussion

In the process of tumor development, the biological behavior is affected by many factors; the tumor–host immune response constitutes the most important part, which dynamically affects tumor progression (23). This study firstly investigated the relationship between the TILs and tumor INF as well as the tumor origin site in GC. TILs are the major effectors encountering malignancy in the frontier; functional phenotypes of lymphocytes have profoundly facilitated the exploration of TILs subsets *in situ*. Various combinations of the TILs subpopulation detection panels have been reported. An international consortium was initiated with the support of the Society for Immunotherapy of Cancer to assess the prognostic value of total tumor-infiltrating T cell counts and cytotoxic tumor-infiltrating T cell counts with the consensus immunoscore assay in patients with stages I–III colon cancer, and the densities of CD3+ and cytotoxic CD8+ T cells in the tumor and the invasive margin were quantified by digital pathology (24). Foxp3+ regulatory cells (Tregs), playing a critical role in immune tolerance and deficiency of anti-tumor immunity, were often used as negative antitumor parameters (25, 26). Therefore, the panel of TILs subset in our study contained CD8+ cytotoxic T cell and Foxp3+ Treg, supplemented with activated CTLs (GrB+ T cell) and primed CD4+ T cells (OX40+ T cell, inducing cytokine production and maintaining a normal immune response).

Due to the special location and structures of the PGC, it displays the clinicopathological characteristics of both esophageal and gastric malignancies, as the esophagogastric junction was a very special transitional area from the squamous epithelium to the glandular epithelium, which is rather different from the typical glandular epithelium of the distal stomach. Different epithelial ingredients with different tumorigenesis might lead to discrepant characteristics for PGC and DGC. In line with the many results of previous research (8, 15, 27), our small sample data also indicated that PGC has more vicious biological behaviors and predicted unfavorable outcomes. In a large sample study, PGC showed a significantly higher incidence of undifferentiated cell types than DGC, and in Lauren’s classifications, PGC showed a higher proportion of diffuse-type cells, whereas DGC exhibited more intestinal-type cells, which was consistent with our study (15). A new finding in our investigation was that PGC frequently has an aggressive infiltration pattern and less number of TILs, especially for the activated anti-tumor cytotoxic lymphocytes (GrB+T). This malignant growth pattern and unfavorable immune microenvironment might inevitably lead to a stronger growth advantage. The predominance of obesity, tobacco abuse, and gastroesophageal reflux disease were reported to be associated with the occurrence of PGC (28–30), which are different from those of DGC, arguing for the different pathogenesis pathways in PGC and DGC. Indeed it is indicated that cancers of the cardia are more frequently associated with deeper gastric wall infiltration, lymph node involvement, and lymphatic vessel invasion (31), of which it is hypothesized that PGC may possess an aggressive biological behavior as the tumor with INFc type grows, all of which may be related to the differences in the pathophysiology of PGC and DGC, while the specific relationship and the mechanism between them need to be further studied.

Additionally, we found tumors with INFc that were associated with a reduced number of CD8+, GrB+T cells, and the whole TILs infiltration. Tumors in proximal sites of the stomach are prone to growing with an infiltrative pattern and infiltrating a fewer number of TILs. Despite that, except for the GrB+ T cell, the other three types of TILs and the total number of TILs were observed to have no statistical differences, but the trends of TILs distribution are obviously shown. It may be attributed to the small size of the tumor in the proximal site, as only 38 cases were PGC among the 147 cases in our study. Anyway, our analytical perspective can open up a new study trace for relative follow-up research. Although INF can be easily determined by routine H&E staining, it has not gained widespread attraction in diagnostic pathology. In the present study, we focused on the correlation between the subsets of TILs and the INF type as well as the association of tumor sites in GC. Tumors with INFc often have a smaller number of TILs compared with INFa or INFb, especially for cytotoxic T cells (CD8+) and activated cytotoxic T cells (GrB+), which are crucial components of antitumor immunity. The current paradigm in tumor immunity suggests that a large number of activated CD8+ effector T cells should be able to attack the tumor cells (32, 33). Moreover, it has been reported that Treg cells can exert an immunosuppressive function so as to limit an effective anti-tumor immune response (26). However, we did not find any significant relation between Foxp3+T cells as well as OX40+T cells and the tumor growth patterns in our study. Additionally, we also compared the immune status between INFa and INFb, while no statistical difference was observed between them, from which it might be concluded that INFa and INFb have a similar immune state. It could also reasonably explain why the investigators always put INFa and INFb into one group and compare them with INFc, but they never give any explanation in their reports. Thereby, we also put INFa and INFb in one group in the subsequent analysis. Similar results were obtained. Compared with the INFa and INFb groups, the INFc type was significantly associated with a shorter overall survival time, and it was strikingly associated with female patients, bigger tumor size, proximal tumor location, and positive lymph node metastasis—a higher number of positive lymph nodes, a much later TNM stage as well as a diffuse type of Lauren classification are suggestive of a more aggressive nature. GC with INFc plus a weak immune defense may be more likely to allow cancer cells to penetrate through the gastric wall and be shed into the surrounding tissue.

Interestingly, tumors in proximal sites were strongly associated with the growth pattern of INFc type. Meanwhile, INFc and the total number of TILs were identified as independent predictive factors for the prognosis of GC in our study. Moreover, in the tumor tissue with INFb, both well-differentiated and poorly differentiated areas exist in the same tissue section, and we found that the density and the sub-type of TILs infiltration were distributed significantly different in disparate differentiated areas, suggesting that tumor cells with different differentiation grades have distinct immunogenicity, resulting in a discrepancy in the type and number of recruited lymphocytes, and could form its special TME under the same immunological background. Despite that, the immune infiltrates are found to be heterogeneous between tumor types and patients, and their effect on prognosis varies in different cancers (34). Our findings reveal a certain relationship between INF and tumor originating site as well as TILs. As the local interactions between the TILs and tumor cells are complex, the specific mechanism and the other relationship remained to be studied in a follow-up work.

## Conclusions

Our study found that GC with an aggressive growth pattern (INFc) originating from the proximal sites (PGC) was always associated with a weak immune response and resulted in a poor prognosis. It opens up a new perspective for research on the biological behavior of the tumor. However, the interaction between them and their synergistic or antagonistic effects in the development of tumors need to be further studied.

## Data availability statement

The original contributions presented in the study are included in the article/supplementary material. Further inquiries can be directed to the corresponding authors.

## Ethics statement

This study was reviewed and approved by the First Affiliated Hospital of Xi’an Jiaotong University (no. XJTU1AF2018LSK-292). The patients/participants provided their written informed consent to participate in this study.

## Author contributions

NZ designed and performed the experiments, analyzed the experimental results, and drafted the manuscript. DW and XH helped to perform the experiments and analyzed the results. NZ and GZ read all the tissue sections. ZqL and YZ helped collected the samples and sorted the related information. ZjL and YW edited and reviewed the manuscript. All authors contributed to the article and approved the submitted version.

## Funding

This study was supported by the Institutional Foundation of The First Affiliated Hospital of Xi’an Jiaotong University (no. 2021QN-23) and the Project of Shaanxi Province Natural Science Basic Research (2022JQ-914).

## Conflict of interest

The authors declare that the research was conducted in the absence of any commercial or financial relationships that could be construed as a potential conflict of interest.

## Publisher’s note

All claims expressed in this article are solely those of the authors and do not necessarily represent those of their affiliated organizations, or those of the publisher, the editors and the reviewers. Any product that may be evaluated in this article, or claim that may be made by its manufacturer, is not guaranteed or endorsed by the publisher.
